# A long-lasting porcine model of ARDS caused by pneumonia and ventilator-induced lung injury

**DOI:** 10.1186/s13054-023-04512-8

**Published:** 2023-06-16

**Authors:** Enric Barbeta, Marta Arrieta, Ana Motos, Joaquim Bobi, Hua Yang, Minlan Yang, Giacomo Tanzella, Pierluigi Di Ginnatale, Stefano Nogas, Carmen Rosa Vargas, Roberto Cabrera, Denise Battaglini, Andrea Meli, Kasra Kiarostami, Nil Vázquez, Laia Fernández-Barat, Montserrat Rigol, Ricard Mellado-Artigas, Gerard Frigola, Marta Camprubí-Rimblas, Pau Ferrer, Daniel Martinez, Antonio Artigas, Carlos Ferrando, Miquel Ferrer, Antoni Torres

**Affiliations:** 1 Surgical Intensive Care Unit, Hospital Clínic de Barcelona, Barcelona, Spain; 2 CIBER de Enfermedades Respiratorias (CIBERES), Instituto de Salud Carlos III, Madrid, Spain; 3 Institut d’Investigacions Biomèdiques August Pi I Sunyer (IDIBAPS), Barcelona, Spain; 4 University of Barcelona (UB), Barcelona, Spain; 5 Department of Cardiology, Erasmus MC, University Medical Center Rotterdam, 3015 Rotterdam, The Netherlands; 6 Department of Respiratory and Critical Care Medicine, Beijing Chao-Yang Hospital, Capital Medical University, Beijing Institute of Respiratory Medicine, Beijing, China; 7 Department of Infectious Diseases, Beijing Chao-Yang Hospital, Capital Medical University, Beijing, China; 8 Department of Anesthesiology, Critical Care Medicine and Emergency, SS. Annunziata Hospital, Chieti, Italy; 9 Department of Anesthesia and Intensive Care, IRCCS for Oncology and Neurosciences, San Martino Policlinico Hospital, Genoa, Italy; 10 Pneumology Service, Respiratory Institute, Hospital Clinic of Barcelona, Villarroel st. 170, 08036 Barcelona, Spain; 11 Department of Anesthesia and Intensive Care, Fondazione IRCCS Ca’Granda Ospedale Maggiore Policlinico, Milan, Italy; 12 Cardiology Department, Institute Clinic Cardiovascular (ICCV), Hospital Clinic, Barcelona, Spain; 13 Department of Pathology, Hospital Clinic, Barcelona, Spain; 14 Critical Care Center, Parc Taulí Hospital Universitari, Institut d’Investigació i Innovació Parc Taulí (I3PT), Universitat Autònoma de Barcelona, Sabadell, Spain; 15 Universitat Pompeu Fabra, Barcelona, Spain

**Keywords:** ARDS, Ventilator-induced lung injury, Porcine model, Pneumonia, Double hit, Injurious mechanical ventilation

## Abstract

**Background:**

Animal models of acute respiratory distress syndrome (ARDS) do not completely resemble human ARDS, struggling translational research. We aimed to characterize a porcine model of ARDS induced by pneumonia—the most common risk factor in humans—and analyze the additional effect of ventilator-induced lung injury (VILI).

**Methods:**

Bronchoscopy-guided instillation of a multidrug-resistant *Pseudomonas aeruginosa* strain was performed in ten healthy pigs. In six animals (pneumonia-with-VILI group), pulmonary damage was further increased by VILI applied 3 h before instillation and until ARDS was diagnosed by PaO_2_/FiO_2_ < 150 mmHg. Four animals (pneumonia-without-VILI group) were protectively ventilated 3 h before inoculum and thereafter. Gas exchange, respiratory mechanics, hemodynamics, microbiological studies and inflammatory markers were analyzed during the 96-h experiment. During necropsy, lobar samples were also analyzed.

**Results:**

All animals from pneumonia-with-VILI group reached Berlin criteria for ARDS diagnosis until the end of experiment. The mean duration under ARDS diagnosis was 46.8 ± 7.7 h; the lowest PaO_2_/FiO_2_ was 83 ± 5.45 mmHg. The group of pigs that were not subjected to VILI did not meet ARDS criteria, even when presenting with bilateral pneumonia. Animals developing ARDS presented hemodynamic instability as well as severe hypercapnia despite high-minute ventilation. Unlike the pneumonia-without-VILI group, the ARDS animals presented lower static compliance (*p* = 0.011) and increased pulmonary permeability (*p* = 0.013). The highest burden of *P. aeruginosa* was found at pneumonia diagnosis in all animals, as well as a high inflammatory response shown by a release of interleukin (IL)-6 and IL-8. At histological examination, only animals comprising the pneumonia-with-VILI group presented signs consistent with diffuse alveolar damage.

**Conclusions:**

In conclusion, we established an accurate pulmonary sepsis-induced ARDS model.

**Supplementary Information:**

The online version contains supplementary material available at 10.1186/s13054-023-04512-8.

## Background

Acute respiratory distress syndrome (ARDS) is a syndrome characterized by capillary endothelial injury and diffuse alveolar damage secondary of cytokines release after a direct risk factor exposure (i.e., infection, aspiration, major trauma) or an indirect risk factors (i.e., sepsis, blood transfusion, major surgery) [1].

The latest definition (i.e., Berlin definition) [1] describes an acute clinical–radiological syndrome characterized by acute respiratory failure and bilateral pulmonary opacities not fully caused by heart failure, fluid overload or other pulmonary pathologies such as lung tumors or atelectasis. As a result, patients with ARDS require oxygen therapy and mechanical ventilation, which is deliverable either invasively or non-invasively.

Animal experimentation has greatly contributed to generating knowledge about ARDS. It was first done in animals with the application of mechanical ventilation and high tidal volume to promote lung damage before being shown in several randomized clinical trials [2]. The benefits of prone position were initially described in animal experimentation as well [3]. Other concepts related to ventilator-induced lung injury such as pulmonary strain, stress and mechanical power were demonstrated in animals before humans [4, 5]. Paradoxically, though, none of these insightful experiments were performed in animal models of ARDS. They were, however, done in animals without any lung injury.

Reproducing ARDS in animals is challenging. The most common cause of ARDS in humans is pneumonia [6], and its definition is based on a constellation of clinical characteristics (acute respiratory failure) and radiological signs (bilateral pulmonary edema) [1]. However, the definition of ARDS in animal models is rarely supported by a disorder of oxygenation or thoracic imaging. Indeed, most pulmonary insults used in an experimental setting differ substantially from human ARDS physiopathology (i.e., intravenous oleic acid or endotoxin administration, surfactant depletion with saline lavage, etc.) [7, 8]. Therefore, animal models usually present features inconsistent with human ARDS like short duration [7, 9] and a significant rate of reversibility [10]. All these drawbacks hamper research in animal experimentation, as it relates to therapies such as new ventilation and extracorporeal life support strategies and targeted treatments.

In this study, we aimed to characterize a double-hit porcine model of ARDS induced by the most common risk factors identified in humans [6]: pneumonia and ventilator-induced lung injury (VILI). To evaluate pneumonia as a cause of ARDS within the presence and absence of VILI, we studied two animal groups: one that comprised pigs with bilateral pneumonia with a high pulmonary strain and the other (control) with bilateral pneumonia and no significant pulmonary strain. After the Berlin criteria for ARDS were met, we monitored gas exchange, pulmonary mechanics, pulmonary and systemic hemodynamics, and pulmonary and systemic inflammatory markers. We also studied the microbiology and histology of the injured lungs.

## Methods

This is an experimental animal study that aimed to characterize the respiratory, hemodynamic, inflammatory, microbiological and histological features of ARDS caused by a bilateral *Pseudomonas aeruginosa* pneumonia and with/without the application of injurious ventilation (a high or low pulmonary strain).

The study was carried out in 10 Large White Landrace pigs (40–48 kg), subjected to 96 h of mechanical ventilation in the prone position (SERVO-I, Maquet, NJ, USA). The experiment was divided into two time frames: (1) induction and (2) model development. During induction, pigs were challenged with a multidrug-resistant (MDR) strain of *P. aeruginosa* and, based on the study group, mechanically ventilated with protective or injurious settings. Model development began when ARDS diagnosis was established; it concluded once the animal was put down.

**Fig. 1:**
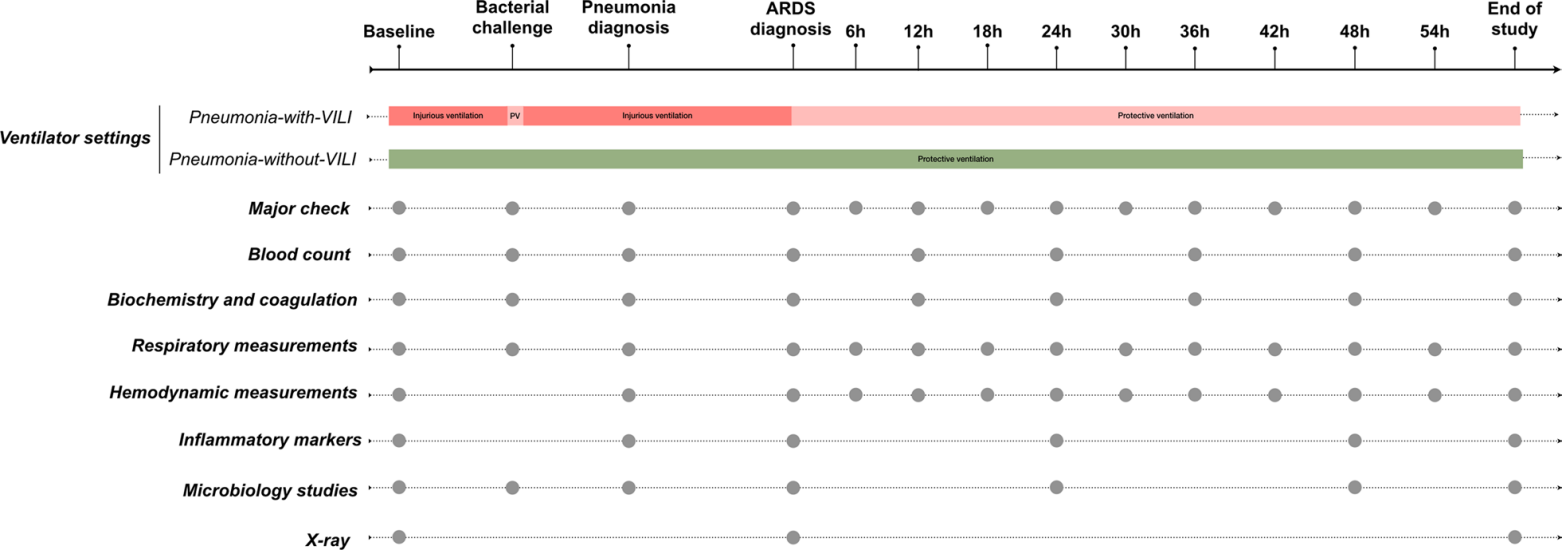
Flowchart of the study. Study assessments are displayed in black dots, while protective and injurious ventilation phases are shown for both study groups. Major check includes: Arterial and mixed venous blood gases, hemodynamics, urine output and bispectral index. Abbreviations: ARDS: acute respiratory distress syndrome; P.V.: protective ventilation. ARDS diagnosis time point in the pneumonia-without-VILI group corresponds to 30 h from bacterial inoculum, which is the median time from inoculum to ARDS diagnosis in the pneumonia-with-VILI group. This equivalence needed to be done for comparison reasons because the pneumonia-without-VILI group never met the Berlin criteria for ARDS diagnosis

As previously described, we prepared the animal and maintained deep anesthesia throughout the experiment [11]. Figure 1 displays main assessments throughout the experiment detailed in Additional file 1.

### Bilateral pneumonia with VILI

This group comprised six animals. Briefly, after animal preparation, we performed 3 h of VILI with a ventilator setting that ensured harmful pulmonary dynamic strain (> 1.5) [12]. Following this, we instilled 15 mL of 10^7^ colony-forming unit (CFU)/mL of MDR *P. aeruginosa,* susceptible to both meropenem and levofloxacin, into each lobe via bronchoscopy (see Additional file 1: Table S1). Injurious mechanical ventilation resumed with the same previous setting, albeit with a positive end-expiratory pressure (PEEP) of 5 cmH_2_O. We diagnosed ARDS when arterial partial pressure of oxygen (PaO_2_)/fraction of inspired oxygen (FiO_2_) < 150 mmHg was achieved. Once criteria for ARDS were met, ventilator mode was changed to volume control (VC) and pigs were ventilated according to a lung protective strategy. Tidal volume was set to aim for normocapnia with a DP that did not exceed 15 cmH_2_O [13].

### Bilateral pneumonia without VILI

The control arm included four animals which presented bilateral pneumonia and received treatment with protective ventilator parameters (no significant strain). Like the previous group, 3 h of ventilation was applied before the bacterial challenge, with a setting that assured a non-harmful pulmonary strain (< 1.5) [12]. After that, we instilled 15 mL of 10^7^ CFU/mL of MDR *P. aeruginosa* into each pulmonary lobe. We continued applying protective ventilation until the end of the experiment. Apart from a DP < 15 cmH_2_O [13], we maintained the thresholds for the most important VILI parameters under a safe range per literature [5, 12, 13] (Table 1).

**Table 1:** Induction and model development periods

	Pneumonia with VILI					Pneumonia without VILI				
	Injurious ventilation	*P. aeruginosa* inoculum	Injurious ventilation (inoculum to pneumonia diagnosis)	Injurious ventilation (pneumonia diagnosis to ARDS diagnosis)	ARDS (to the end of the experiment)	Protective ventilation	*P. aeruginosa* inoculum	Protective ventilation (inoculum to pneumonia diagnosis)	Pneumonia (to the end of the experiment)	ARDS
Duration (h)	3.1 ± 0.1	0.9 ± 0.1	12.0 ± 0.7	16.0 ± 2.3	46.8 ± 7.7	3.1 ± 0.2	NA	7.2 ± 1.1	82.4 ± 1.2	NA
FiO2 (%)	100.0 ± 0.0	100.0 ± 0.0	100.0 ± 0.0	100.0 ± 0.0	100.0 ± 0.0	41.3 ± 0.0	NA	40.6 ± 0.6	44.4 ± 1.1	NA
TV (mL/kg)	33.3 ± 2.4	10.5 ± 0.0	27.5 ± 1.6	25.1 ± 0.9	5.7 ± 0.2	8.1 ± 0	NA	7.8 ± 0.2	7.2 ± 0.1	NA
RR (breaths/min)	12.3 ± 0.0	20.0 ± 0.0	11.3 ± 0.9	10 ± 0.0	45.8 ± 1.0	15.4 ± 0.4	NA	16.8 ± 0.1	23.7 ± 1.1	NA
PEEP (cmH_2_O)	0.0 ± 0.0	3.0 ± 0.0	4.9 ± 0.0	4.9 ± 0.1	9.5 ± 0.3	4.0 ± 0.0	NA	4.3 ± 0.0	4.8 ± 0.2	NA
DP (mmHg)	27.7 ± 0.2	12.5 ± 0.0	30.1 ± 0.3	29.9 ± 0.2	17.7 ± 0.5	9.5 ± 0.5	NA	12.3 ± 0.0	13.5 ± 0.4	NA
MP	NA	10.5 ± 1.6	34.7 ± 0.8	28.35 ± 1.2	26.9 ± 1.7	5.23 ± 0.7	NA	6.65 ± 1.0	10.1 ± 0.3	NA
Ventilatory mode	PC	VC	PC	PC	VC	VC	NA	VC	VC	NA

After the inoculum, a short period of protective ventilation was performed to ease bacteria engraftment. Note that this was not necessary in the other group because protective ventilation was already ongoing. Data are reported as mean ± standard error

*ARDS* acute respiratory distress syndrome, *DP* driving pressure, *FiO*_*2*_ fraction of inspired oxygen, *MP* mechanical power, *P. aeruginosa, Pseudomonas aeruginosa*, *PC* pressure control, *PEEP* positive end-expiratory pressure, *RR* respiratory rate, *TV* tidal volume, *VC* volume control

### Pneumonia diagnosis

Pneumonia was clinically diagnosed in both groups based on a decline in PaO_2_ and one of the following signs of infection: body temperature > 38.5 °C, a white blood count > 14·10^9^/L and purulent secretions [14]. At pneumonia diagnosis, we started antimicrobial therapy with 25 mg/kg of meropenem every 8 h and 10 mg/kg of levofloxacin every 24 h, as dual therapy recommended by international guidelines [15].

### Statistical analysis

The results were reported as the mean and standard error of the mean. Categorical variables were presented as umbers and percentage. For continuous variables, we compared groups and over time using the repeated measure two-way ANOVA test. Bonferroni’s post hoc multiple comparison was used to control the experiment-wise error rate. Comparison between groups at a fixed time point was assessed using the Mann–Whitney nonparametric test. Either the Chi-square test or Fisher’s exact test was used to compare categorical variables. A two-sided *p* value < 0.05 was considered statistically significant. All statistical analyses were performed using the GraphPad Prism 8 (version 8.0.1 (244), San Diego, CA, USA).

## Results

Ten animals (42.9 ± 1.13 kg) were divided into two groups: six animals comprised the pneumonia-with-VILI group and four animals the pneumonia-without-VILI group. In the former group, two pigs were put down before the end of the experiment due to severe respiratory failure and severe respiratory acidosis. The mortality rate reached 33.3%. All animals in the pneumonia-without-VILI group completed the study.

### Induction period in pigs with pneumonia according to the presence or absence of VILI

**Fig. 2:**
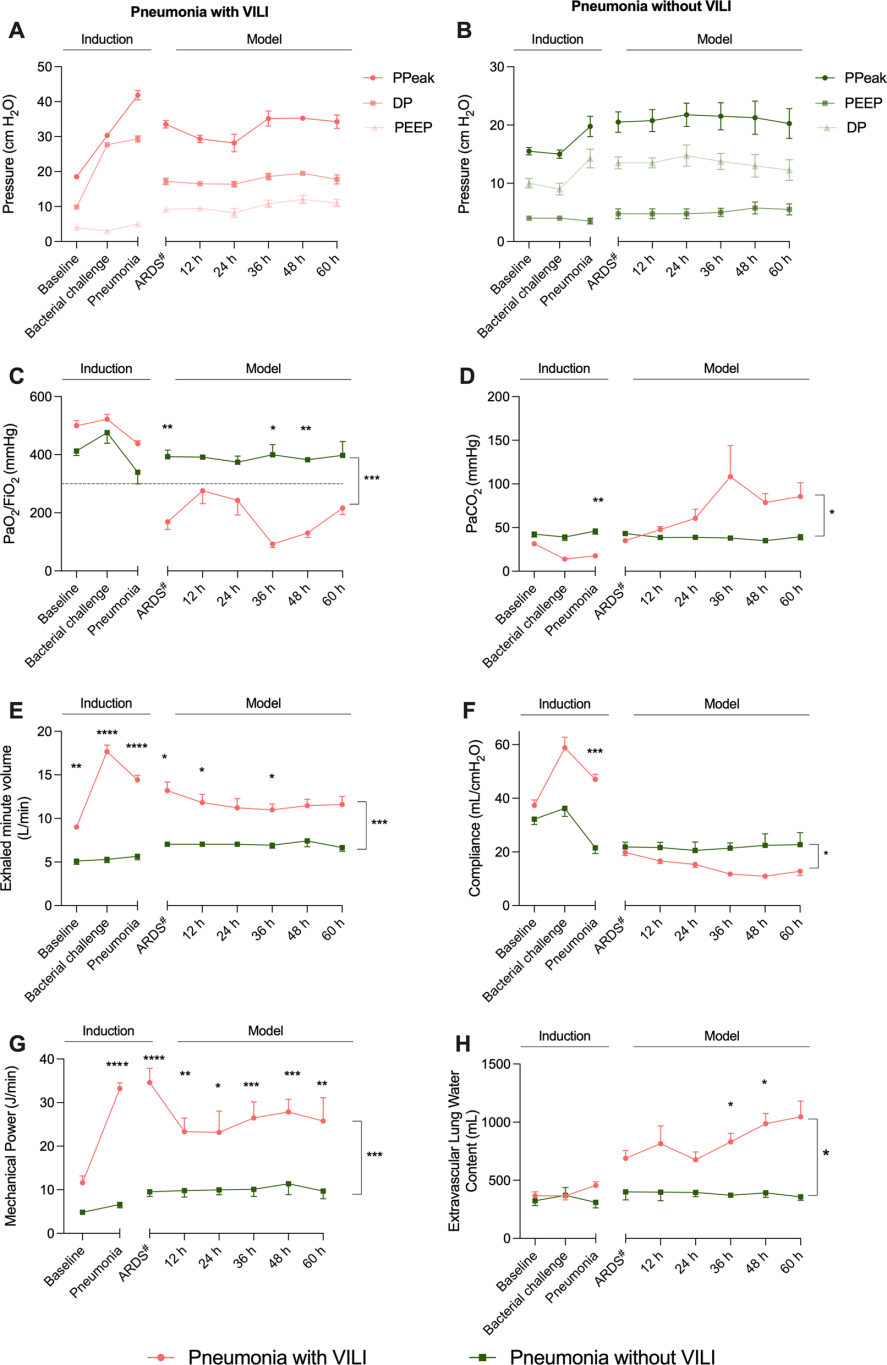
Respiratory function. Pink lines and dots represent animals from the pneumonia-with-VILI group, and the green ones represent those animals from the pneumonia-without-VILI group. **A** Evolution of airway pressures in the pneumonia-with-VILI group. **B** Evolution of airway pressures in the pneumonia-without-VILI group. **C** Evolution of PaO_2_/FiO_2_ throughout the study with periods of moderate and severe respiratory failure in the pneumonia-with-VILI group. Note that in the group of pigs without VILI, it does not decrease below 300 mmHg. **D**, **E** PaCO_2_ and exhaled minute volume were increased in the group of pneumonia with VILI after ARDS diagnosis and were stable in animals that were ventilated protectively. Note the high variability in PaCO_2_ at 36 h of ARDS diagnosis is due to the death of two animals. **F** Higher compliance was observed in the pneumonia-with-VILI model during induction period, whereas after ARDS diagnosis, it was lower. **G** A significantly higher mechanical power was observed between groups and at every time point. **H** Extravascular lung water increased in both groups throughout the study time points, showing evidence of an increase in lung permeability. However, in the pneumonia-with-VILI group, it was significantly higher. Abbreviations: ARDS: acute respiratory distress syndrome; DP: driving pressure; PEEP: positive end-expiratory pressure; Ppeak: peak pressure; VILI: ventilator-induced lung injury. Data are reported as mean ± standard error of mean (SEM). # ARDS diagnosis time point in the pneumonia-without-VILI group corresponds to 30 h from bacterial inoculum, which is the median time from inoculum to ARDS diagnosis in the pneumonia-with-VILI group. This equivalence needed to be done for comparison reasons because the pneumonia-without-VILI group never met the Berlin criteria for ARDS diagnosis. *p < 0.05, **p < 0.01, ***p < 0.001, ****p < 0.0001

Table 1 and Fig. 2 display parameters related to mechanical ventilation, gas exchange and respiratory mechanics observed during the induction period. During this period, we performed a 15-mL *P. aeruginosa* challenge of 7.77 ± 0.11 log CFU/mL in each pulmonary lobe in both groups.

After the bacterial challenge, we continued injurious ventilation in the VILI group for 28.04 ± 3.03 h until the Berlin criteria were met, with the mean PaO_2_/FiO_2_ being 129.62 ± 6.1 mmHg at ARDS diagnosis. Bilateral opacities were identified by X-ray in all six pigs. In contrast, the pneumonia-without-VILI group never met the Berlin criteria for ARDS. PaO_2_/FiO_2_ did not decrease below 300 mmHg at any time point (mean PaO_2_/FiO_2_ was 389.90 ± 3.97 mmHg) (*p* < 0.0002) (Fig. 2).

**Fig. 3:**
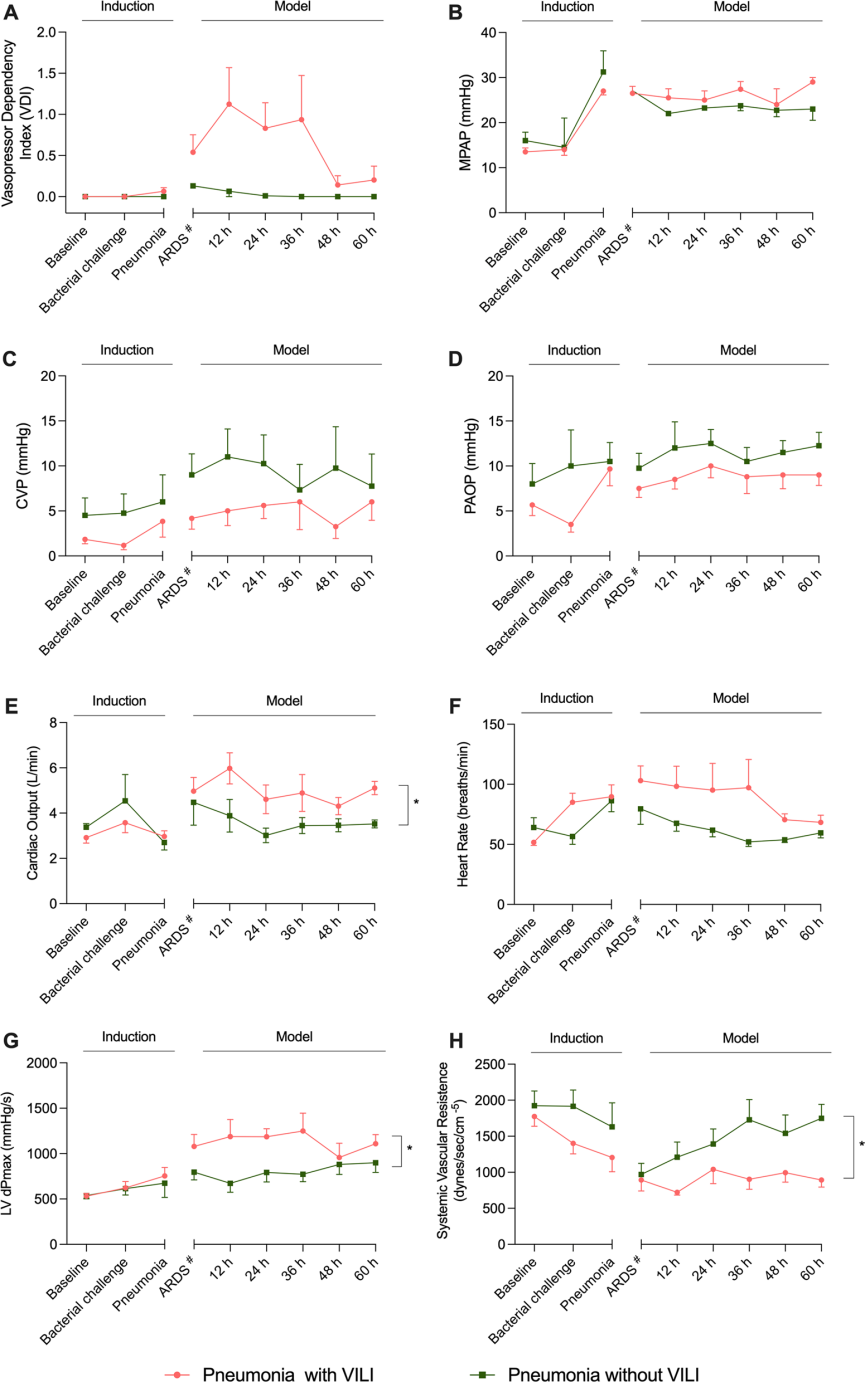
Hemodynamics. Pink lines and dots represent animals from the pneumonia-with-VILI group, and the green ones represent those animals from pneumonia-without-VILI group. **A** The evolution of VDI showed the need to use norepinephrine to maintain mean arterial pressure in the group of pigs with VILI and in those pigs ventilated protectively. **B** Mean pulmonary pressure increased at pneumonia diagnosis and remained stable in the pneumonia-with-VILI group, whereas it decreased in the group without VILI. **C**, **D** CVP and PAOP did not present significant differences between groups. **E**, **F** A higher cardiac output and heart rate were found in animals with VILI in comparison with those ventilated protectively. **G** Animals developing ARDS presented higher maximal left ventricular pressure rise (LV dPmax) as a marker of systolic function. **H** Systemic vascular resistance decreased in animals with pneumonia and VILI in comparison with the group of pigs without VILI. Abbreviations: ARDS: acute respiratory distress syndrome; CVP: central venous pressure; LV dPmax: maximal left ventricle pressure rise; MAP: mean arterial pressure; PAOP: pulmonary arterial occlusion pressure; VDI: vasopressor dependency index. Data are reported as mean ± standard error of mean (SEM). # ARDS diagnosis time point in the pneumonia-without-VILI group corresponds to 30 h from inoculum, which is the median time from inoculum to ARDS diagnosis in the pneumonia-with-VILI group. This equivalence needed to be done for comparison reasons because the pneumonia-without-VILI group never met the Berlin criteria for ARDS diagnosis. *p < 0.05

No significant differences were found in the hemodynamic parameters between groups during the induction period, as shown in Fig. 3.

**Fig. 4:**
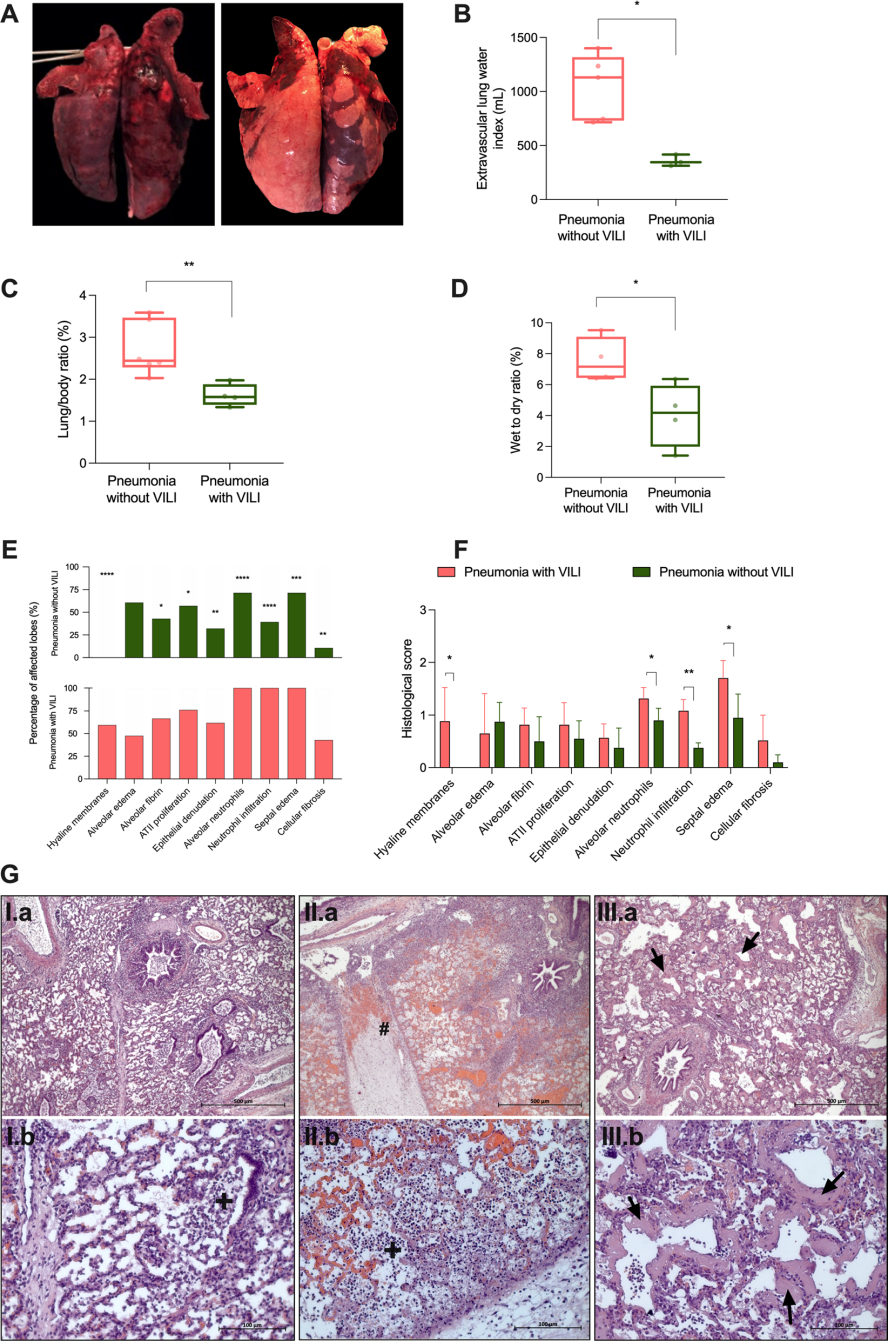
Lung findings. Pink lines and dots represent animals from the pneumonia-with-VILI group, and the green ones represent those animals from the pneumonia-without-VILI group. **A** Macroscopic findings: on the left, non-dependent images of an animal from the pneumonia-with-VILI group and on the right, from the pneumonia-without-VILI group. **B** Significantly higher EVLW in the pneumonia-with-VILI group at the end of the study (*p* = 0.02). **C** Lung weight-to-body weight ratio was significantly higher in animals with pneumonia and VILI (*p* = 0.009). **D** The wet lung weight-to-dry lung weight ratio was significantly higher in the pneumonia-with-VILI group (*p* = 0.029). **E** Percentage of lobes affected with histological features in both study groups. **F** Histological score evaluated in both groups (0–3). **G** I: Lung tissue microscopic findings in pneumonia-without-VILI animals presenting inflammatory neutrophilic infiltrate ( +). **G** II and III: Lung tissue microscopic findings in pneumonia-with-VILI animals showed moderate neutrophil infiltration of the alveolar space and septae, severe edema and hemorrhage, as well as neutrophil infiltration ( +), severe thickening of interlobular septae (#) and evident presence of hyaline membranes (black arrows) surrounding alveoli. Abbreviations: ARDS: acute respiratory distress syndrome; ATII: alveolar epithelial type II cells; VILI: ventilator-induced lung injury. *p < 0.05, **p < 0.01, ***p < 0.001, ****p < 0.0001

Pneumonia was clinically and microbiology established at 11.99 ± 0.67 and 7.25 ± 1.12 h post-inoculum in the pneumonia-with-and-without-VILI groups, respectively (*p* = 0.01). Additional file 1: Table S2 shows the achieved criteria of pneumonia diagnosis. No differences in *P. aeruginosa* burden were observed between study groups at pneumonia diagnosis, neither in tracheal secretions (*p* = 0.46) nor in BAL samples (*p* = 0.57) (Fig. 4).

### Model development: respiratory function

Animals comprising the pneumonia-with-VILI group maintained a PaO_2_/FiO_2_ below 300 mmHg until the end of experiment (model development period). The mean duration of an ARDS diagnosis in animals that survived was 58.7 ± 1.3 h. For those who were killed due to clinical instability, it was 29.9 h and 16.3 h each. During this period (model development), the mean PaO_2_/FiO_2_ was 187.80 ± 28.46 mmHg, with the lowest measure recorded at 83.00 ± 5.45 mmHg. Figure 2 displays the evolution of respiratory system mechanics during model development. Respiratory system compliance was lower in animals with VILI compared with those without VILI (14.54 ± 1.30 and 21.77 ± 0.32 cmH_2_O/L) (*p* = 0.011). Furthermore, DP and mechanical power showed significant differences between groups (*p* = 0.011 and *p* = 0.001, respectively). Mean values were 17.65 ± 0.50 cmH_2_O and 26.87 ± 1.72 J/min in the group of pneumonia with VILI and 13.46 ± 0.34 cmH_2_O and 10.09 ± 0.27 J/min in the group of pneumonia without VILI. After the ARDS diagnosis time point, PaCO_2_ in animals with VILI was significantly higher than in those without VILI (69.28 ± 10.95 and 38.85 ± 1.076 mmHg, *p* = 0.027). This also was observed in exhaled minute volume (11.71 ± 0.32 and 7.02 ± 0.10 L/min, *p* < 0.001). Furthermore, mean EVLW was 840.2 ± 61.73 mL/kg in the pneumonia-with-VILI group and 385.6 ± 6.90 mL/kg in the pneumonia-without-VILI group (*p* = 0.013), as shown in Fig. 2. Ventilator setting adjustments are displayed in Additional file 1: Fig. S1.

### Model development: systemic and pulmonary hemodynamics

Mean pulmonary pressure increased in pigs with ARDS: Mean values were 26.23 ± 0.73 mmHg compared with 22.79 ± 0.28 mmHg in in the pneumonia-without-VILI group (*p* = 0.056). Mean arterial pressure in pigs with ARDS remained at 72.48 ± 1.23 mmHg. In the other group, it was 84.79 ± 5.21 mmHg (*p* = 0.003). Pigs from the pneumonia-with-VILI group presented a higher vasopressor dependency index (VDI); however, it was not significant (0.63 ± 0.16 vs 0.03 ± 0.02, *p* = 0.158). After an ARDS diagnosis, systemic vascular resistances were lower in the pneumonia-with-VILI group than in the pneumonia-without-VILI group (907.3 ± 44.76 dynes/s/cm^−5^ 1432.71 ± 124.41 dynes/s/cm^−5^; *p* = 0.02). Figure 3 shows other hemodynamic parameters. Laboratory findings and fluid balance are reported in Additional file 1: Table S3 and Fig. S2.

### Imaging, necropsy and histology

We performed X-ray images of two different projections at baseline and the end of the experiment. While normal lung appearance was found at baseline, bilateral opacities were observed at the end of the experiment in both groups. X-ray images were also performed at ARDS diagnosis in animals with VILI, revealing bilateral opacities too. At necropsy, macroscopic findings were more severe in those with VILI than in animals presenting pneumonia without VILI, as shown in Additional file 1: Fig. S3.

**Fig. 5:**
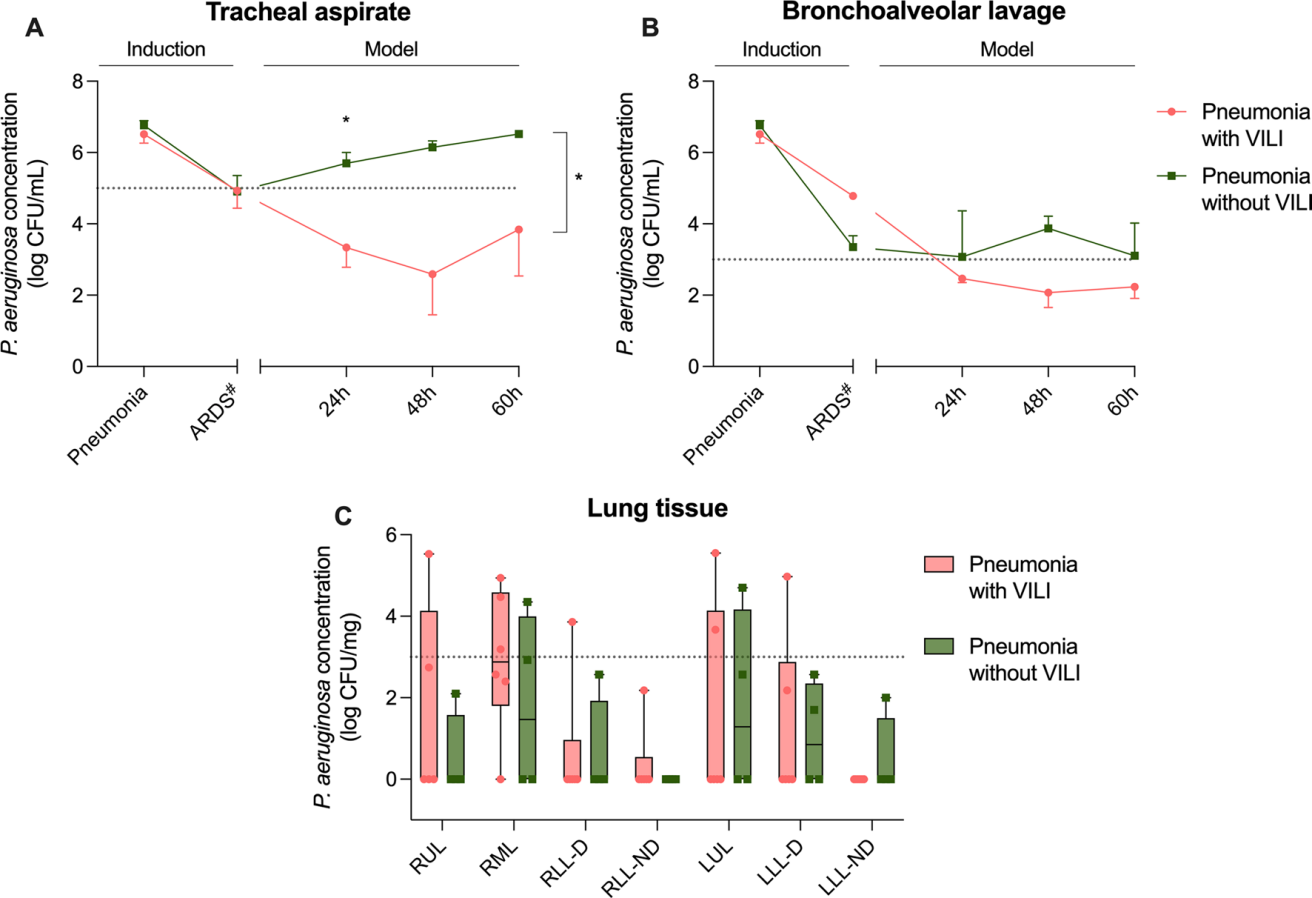
Microbiology assessments. Pink lines, dots and boxes represent animals from the pneumonia-with-VILI group, and the green ones represent those animals from the pneumonia-without-VILI group. **A** After pneumonia diagnosis and antibiotic start, *Pseudomonas aeruginosa* concentration in tracheal secretions decreased (*p* = 0.007), more evident in animals with VILI. **B** With respect to BAL fluids, the burden decreased throughout the study (*p* = 0.001). **C** In lung tissue, mean values resulted below 3 log CFU/mL after having received 8.2 ± 0.9, 3 ± 0.4 and 7 ± 0.7 doses of meropenem, levofloxacin and ceftriaxone, respectively. Abbreviations: ARDS: acute respiratory distress syndrome; CFU: colony-forming units; VILI: ventilator-induced lung injury. Pulmonary lobes: RUL, right upper lobe; RML, right medium lobe; RLL-D, right lower lobe-dependent; RLL-ND, right lower lobe-non-dependent; LUL, left upper lobe; LLL-D, left lower lobe-dependent; LLL-ND, left lower lobe-non-dependent. Data are reported as mean ± standard error. # ARDS diagnosis time point in the pneumonia-without-VILI group corresponds to 30 h from inoculum, which is the median time from inoculum to ARDS diagnosis in the pneumonia-with-VILI group. This equivalence needed to be done for comparison reasons because the pneumonia-without-VILI group never met the Berlin criteria for ARDS diagnosis. *p < 0.05

Figure 5 displays the main lung findings. Macroscopically, all pigs presented with findings consistent with bilateral pneumonia. However, animals that developed ARDS also had widespread hepatization, severe edematous appearance, pleural effusion and adherence. Importantly, in the pneumonia-with-VILI group, we found higher vascular permeability, as revealed by both a higher lung/body weight ratio (2.71 ± 0.26 in the pneumonia-with-VILI group vs. 1.61 ± 0.13% in the pneumonia-without-VILI group, *p* = 0.009) and lung wet-to-dry ratio (7.56 ± 0.73 in the pneumonia-with-VILI group vs. 4.03 ± 1.03% in the pneumonia-without-VILI group, *p* = 0.029).

In the pneumonia-with-VILI group, all except one (83%) presented hyaline membrane formation consistent with diffuse alveolar damage (DAD). However, DAD and hyaline membranes were absent in all the animals in which pneumonia was induced without VILI. Furthermore, when compared with the pneumonia-without-VILI group, animals that developed ARDS also presented a rise in intra-alveolar (*p* = 0.033) and interstitial neutrophil infiltration (*p* = 0.005), as well as increased septal edema (*p* = 0.038). Alveolar edema, intra-alveolar fibrin and hemorrhage, proliferation of alveolar type II cells and epithelial denudation were reported in both groups without differences. In Additional file 1: Fig. S4, the same features are displayed by dependent and non-dependent areas.

### Systemic and local inflammatory markers

As shown in Additional file 1: Fig. S5, systemic IL-6 and IL-8 had significant time-dependent changes (*p* < 0.0001 and *p* = 0.024). At pneumonia diagnosis, plasma IL-6 was higher in the pneumonia-with-VILI group (579.94 ± 138.79 vs. 356.79 ± 233.66 pg/mL, *p* = 0.038). However, after the ARDS diagnosis (model development period), these differences disappeared. IL-6 and IL-8 also presented an increase in BAL, albeit without significant differences between groups.

### Microbiology assessments

Figure 5 displays the main microbiological results. Throughout the model development period, animals from the pneumonia-with-VILI group presented a lower burden of *P. aeruginosa* in tracheal aspirates (4.24 ± 0.68 log CFU/mL in the pneumonia-with-VILI group and 6.01 ± 0.32 log CFU/mL in the pneumonia-without-VILI group, *p* = 0.027). However, it was not different for lung tissue or BAL. At the end of the experiment, tracheal aspirate cultures from both groups tested > 3 log CFU/mL. In lung tissue, mean values were below 3 log CFU/mL in both groups.

## Discussion

We developed a model of pulmonary sepsis-induced ARDS in pigs that had long-lasting features consistent with human ARDS. Our model was characterized by the following aspects: (I) development of severe oxygenation disorders; (II) development of hypercapnia despite the application of high-minute ventilation (reflecting an increase in dead-space ventilation); (III) decrease in respiratory system static compliance; (IV) increase in pulmonary permeability; (V) presence of cardiovascular dysfunction (sepsis-induced vasoplegia); (VI) pulmonary and systemic inflammation; and (VII) signs of diffuse alveolar damage.

The model presented fulfills recent recommendations for animal experimentation in the field of acute lung injury [16]. That considered, we demonstrated histological evidence of tissue injury; alteration of the alveolar–capillary barrier; presence of systemic and pulmonary inflammatory response; and severe respiratory dysfunction. Furthermore, other relevant characteristics specific to the model should be underscored: First and foremost, the pulmonary insult used in the induction period is the main cause of ARDS in humans (bacterial pneumonia). Moreover, the second hit used in the model (VILI) might be unavoidable in human ARDS, as ventilation with high strain and stress is needed to maintain gas exchange in the most severe patients [6]. Second, the large duration of the experiment allows for tracking changes in respiratory physiology. Third, large animals allow for advanced monitoring (i.e., electrical impedance tomography, and analysis of chest wall and pulmonary respiratory mechanics, pulmonary and systemic hemodynamics, etc.) as well as evaluation of complex therapies such as extracorporeal membrane oxygenation and mechanical ventilation.

### Comparison of animal models and their characteristics

Given the clinically relevant insults used (pneumonia and VILI) as well as the physiological characteristics exhibited during the 96-h experiment period, we believe that the model presented has higher accuracy in reproducing human ARDS compared with others [7, 8, 16, 17].

In line with our study, the repeated lavage model presents remarkable gas exchange disorders and diminished compliance due to surfactant depletion. Specifically, our ARDS model shows a mean PaO_2_/FiO_2_ which, throughout the model development, ranges in moderate severity with periods of severe instability. Moreover, notably diminished and progressively deteriorating compliance is found throughout the model development period as well. Surfactant depletion is not the main mechanism of respiratory failure in adult ARDS; hence, this model fails to reproduce other clinical features of ARDS. For example, in contrast from our model, only modest epithelial injury signs are present; permeability changes, scarce; and clinical features described, reversible, lasting less than 24 h [9, 10, 18]. Our model, however, presents a histology that demonstrates that severe epithelial damage and disorders in oxygenation are maintained up to approximately 60 h. In the surfactant depletion model, some authors reported an increase in BAL cytokines, but the role of inflammation is unclear [9]. We demonstrated, nonetheless, an increase in both systemic and pulmonary inflammation and signs of sepsis-associated cardiovascular dysfunction.

Similar flaws are identified in the oleic acid (OA) model. This one mimic ARDS caused by fat embolism, an exceptional etiology of syndrome [10]. In the OA model, the pathophysiology of pulmonary injury is unknown and unlikely similar to the most common causes of ARDS such as pneumonia, the risk factor used in our model [6]. OA administration raises pulmonary arterial pressure and intrapulmonary shunt [19–21] and produces a marked increase in EVLW [20]. We also showed a rise in the mean pulmonary arterial pressure and EVLW. We tracked changes in PaO_2_/FiO_2_ as surrogate of pulmonary shunt, identifying periods with a value below 100 mmHg (i.e., severe ARDS). In the OA model, an inflammatory response is shown with an increase in some inflammatory markers in lung such as IL-6 and IL-1 beta [22–24]. At histological examination, however, only mild signs of lung injury are found with no hyaline membrane formation [20, 23, 25]. In our model, though, we demonstrated DAD in 83% of histological specimens of pigs with ARDS. In the OA model, the severity of lung injury is somewhat unpredictable because animals present different responses per OA doses and sudden, intense hemodynamic instability during infusion [23, 25]. Like the repeated lavage model, the oleic acid-induced lung injury is also reversible within several hours, contrasting with our 60-h ARDS period [21, 23].

Systemic administration of bacterial lipopolysaccharides (LPS) was one of the earliest approaches used to study sepsis-induced ARDS. LPS administration markedly decreases PaO_2_ and systemic arterial pressure. It also provides important information about host inflammatory responses in BAL and serum [7, 26]. For that reason, it is a good choice for pathophysiological studies of infection and ARDS [7, 26]. Our model follows this approach; however, we instill viable bacteria—rather than bacterial toxins—into the lungs, thus fulfilling the complete clinical picture of severe pneumonia. During the model development, a high burden of *P. aeruginosa* was found in BAL and tracheal aspirates. While we did not find differences in BAL samples, we did identify a lower bacterial burden in tracheal aspirates from animals with ARDS in comparison with those who did not develop it. The interpretation of this finding is not clear. We speculate that the plasma leak into the lungs of pigs with ARDS prompted tracheal aspirations more frequently and promoted bacterial clearance. Histological alterations observed in the LPS model are mainly neutrophil parenchymal infiltration and mild edema, features that resemble those seen in pneumonia. However, in this model, hyaline membranes are lacking [7, 27], suggesting milder epithelial damage and lower pulmonary permeability compared with our model. Other limitations of this model are short duration, significant differences in endotoxin response between species and variability in biological activity of endotoxin preparations [8]. In contrast, the duration of our model is enough to perform multiple analyses, given its reproducible nature as well.

The smoke/burn is a reproducible model that resembles the clinical time course of ARDS during the first 24 h. It can be used in studies up to 96 h [28]. However, smoke inhalation as etiology of ARDS is not frequent, and the pathophysiology of lung injury is different from sepsis [6]. Although some studies achieve severe respiratory failure [28], this does not last longer 24 h [29, 30]. Inflammatory markers as IL-6 are found elevated in BAL and plasma [28, 31]. Histology observations demonstrate a presence of inflammatory infiltrate, mild hemorrhage and edema, albeit no hyaline membranes [28, 31, 32].

Injurious ventilation has been used as a unique or double-hit model to reproduce ARDS in animals [8, 32]. In rodent animal models, VILI causes an impairment in respiratory mechanics [33, 34], tissue injury [33, 35] and pulmonary permeability [34], but oxygenation disorders are mild and do not usually meet ARDS criteria [36–39]. These findings in small animals are difficult to translate in terms of humans. Contrarily, in larger animal models, VILI provokes severe respiratory failure. Unlike our model, though, it does not last longer than 24 h [38–42]. In these models, inflammation caused by VILI was also evaluated by several methods including cytokine analysis in BAL fluid, plasma and lung tissue, and either immunohistochemistry or immunofluorescence [8, 33-44]. While some studies confirmed that VILI induces a pulmonary inflammatory response [33–39], others did not [44]. These differences might be explained by either the intensity of VILI observed between studies or the primary pulmonary insult performed before aggressive ventilation was started [45]. Interpretations of our findings should consider that we used a DP of up to 30 cmH_2_O to ensure a clinically significant pulmonary strain and that such ventilation was performed in lungs affected by a primary inflammatory insult (pneumonia). Interestingly, as observed in our model, other authors reported the presence of hyaline membranes and diffuse alveolar damage in animals ventilated in an injurious fashion [40].

### The effect of ventilator-induced lung injury in pneumonia

In our model, the group with low pulmonary strain—no VILI—presented oxygen disorders that were mild and did not meet the Berlin criteria for ARDS. On the other hand, pigs comprising the pneumonia-with-VILI group had sustained life-threatening gas exchange disorders, as well as other remarkable disturbances. In the current model, however, we did not find clinically relevant extrapulmonary organ dysfunctions other than hemodynamic failure (sepsis-induced vasoplegia). The latter was more prominent in the pneumonia-with-VILI group. Of note, while pigs with ARDS exhibited a high inflammatory response, this was not clearly influenced by the presence of VILI during model development. Importantly, high mortality (33.3%) found only in the group of pigs ventilated with the high pulmonary strain was driven by respiratory acidosis and refractory respiratory failure.

The findings described between groups merit clinical interpretation. Refractory respiratory failure—either manifested as severe hypoxemia or respiratory acidosis—represents a non-negligible cause of death and morbidity in ARDS [46, 47]. Given the extreme oxygenation and ventilation disorders found in the group of pigs with VILI, maintaining protective ventilation was challenging after the ARDS diagnosis (model development period). This is revealed by an increase in mechanical power throughout all the experiment which, consequently, might have dramatically diminished the chances of pulmonary recovery in the group with VILI. Our results, therefore, suggest that a high pulmonary strain might ease progression to ARDS in pneumonia and perhaps raise respiratory-attributable death.

### Limitations

Some limitations are present in this study. First, as a result of severe respiratory failure, mortality reported in this model was high (33.3%). Second, we only studied ten non-randomized pigs and just those treated with high pulmonary strain fulfilled the diagnostic criteria for ARDS. Nevertheless, the dispersion of data was low, and our findings were consistent. Due to logistical constraints, animals were not randomized. Third, critically ill patients usually present with significant comorbidities that determine prognosis. However, animals used in the model were female, young and healthy. Moreover, the sequence of events in the clinical setting differs, as patients initially experience an insult that results in respiratory insufficiency, followed by the need for mechanical ventilation. In our model, healthy animals underwent injurious ventilation, were then subjected to bacterial challenge and finally continued to receive injurious ventilation. Fourth, *P. aeruginosa* is a cause of pneumonia in patients with specific risk factors such as airway diseases (i.e., chronic obstructive pulmonary disease) and immunosuppression. Fifth, there was not a control group of pigs without pneumonia that received treatment with aggressive ventilation.

## Conclusion

In conclusion, we established an accurate pulmonary sepsis-induced ARDS model. To carry out this model, it is necessary to induce bilateral pneumonia and increase pulmonary damage with VILI. The clinically relevant pulmonary insults and features exhibited, long duration and capacity for invasive procedures and continuous monitoring to be performed constitute the most important elements of this model. For those reasons stated, we believe that this model is appropriate for studying novel lung protective strategies and respiratory rescue therapies for very severe ARDS.

## Supplementary Information

**Additional file 1.** Additional methods, tables and figures.

## Data Availability

The datasets used and/or analyzed during the current study are available from the corresponding author on reasonable request.

## Abbreviations

ARDS: Acute respiratory distress syndrome
BAL: Bronchoalveolar lavage
CFU: Colony-forming unit
CVP: Central venous pressure
DP: Driving pressure
EVLW: Extravascular lung water
FiO_2_: Fraction of inspired oxygen
ICU: Intensive care unit
LV dPmax: Maximal left ventricular pressure rise
MAP: Mean arterial pressure
MDR: Multidrug resistance
OA: Oleic acid
PaO_2_: Partial pressure of oxygen in arterial blood
PAOP: Pulmonary artery occluded pressure
PaCO_2_: Partial pressure of carbon dioxide in arterial blood
PC: Pressure control
PEEP: Positive End-expiratory pressure
RR: Respiratory rate
VC: Volume control
VDI: Vasopressor dependency index
VILI: Ventilatory-induced lung injury
ZEEP: Zero end-expiratory pressure
