# Supplementary material for: A long-lasting porcine model of ARDS caused by pneumonia and ventilator-induced lung injury
Source: Crit Care. 2023 Jun 16;27:239. doi: 10.1186/s13054-023-04512-8 (PMC10276390; doi:10.1186/s13054-023-04512-8)

**A LONG-LASTING PORCINE model of ARDS CAUSED BY PNEUMONIA AND VENTILATOR-INDUCED LUNG INJURY**

**# Co-corresponding authors:**

Ana Motos, PhD

Pneumology Department

Hospital Clinic of Barcelona

Villarroel st. 170

08036 Barcelona (SPAIN)

Voice: 0034 932275400

Email: [amotos@clinic.cat](mailto:amotos@clinic.cat)

Antoni Torres, MD, PhD

Pneumology Department

Hospital Clinic of Barcelona

Villarroel st. 170

08036 Barcelona (SPAIN)

Voice: 0034 932275400

Email: [atorres@clinic.cat](mailto:atorres@clinic.cat)

**ADDITIONAL METHODS**

This study was conducted in the animal facilities located in the University of Barcelona Department of Medicine (Spain). All procedures were done following the European Directive 2010/63/UE and Spanish RD 53/2013 regulations related to the Guide for the Care and Use of Laboratory Animals. The study protocol was approved by the Animal Experimentation Ethics Committee of the University of Barcelona (approval reference number: DTES 10543).

Animal preparation and deep anaesthesia were upheld throughout the experiment, including an ultrasound-guided cannulation of both the femoral artery and jugular vein, and bladder catheterization. Advanced hemodynamics were monitored using PiCCO® (PULSION Medical Systems AG, Munich, Germany) and Swan-Ganz catheters. Also, they were monitored with the bispectral index. In order to prevent endogenous colonization, 1g of ceftriaxone was administered before intubation, followed by 75 mg/kg for a 1-hour infusion every 12 hours.

The induction period started just after surgical preparation and ended when criteria to diagnose moderate-to-severe ARDS were met. Model development began when ARDS diagnosis was established and concluded after the animal was put down. Samples obtained from the main assessments were processed as previously described (1).

Bilateral pneumonia with VILI

This group comprised six pigs, in which pneumonia was induced by a MDR *P. aeruginosa* isolate. Pulmonary damage in these animals was amplified via VILI. During the induction period, we aimed for high driving pressure (DP) as a subrogate parameter of lung strain. After animal preparation, we performed 3h of VILI with the following ventilator settings: pressure control (PC) mode, respiratory rate (RR) 10 rpm, zero end-expiratory pressure (ZEEP), FiO_2_ 100%, I/E 1:2, and inspiratory pressure of 30 cmH2O (corresponding to a DP of 30 cmH_2_O and a baseline tidal volume of 33.3±2.3 mL/kg).

Furthermore, RR was applied after a diagnosis of moderate-to-severe ARDS, aiming for normocapnia and not exceeding 50 rpm. Following a rise in arterial partial pressure of carbon dioxide (PaCO_2_), we increased RR first before tidal volume to perform mechanical ventilation protectively. Ventilator parameters exceeding these limits were needed in several periods of clinical instability, such as severe respiratory acidosis. The inspiratory fraction of oxygen was maintained at 100% throughout the experiment and PEEP was set at high levels (up to 12 cmH_2_O) according to oxygenation.

Bilateral pneumonia without VILI

This group of pigs were ventilated in VC mode, RR 10 bpm, PEEP 4 cmH_2_O, FiO_2_ 100%, I/E 1:2, and a tidal volume of 8mL/Kg that corresponded to a DP of 9.5 cmH_2_O.

For time [standardization](https://www.linguee.es/ingles-espanol/traduccion/standardization.html) reasons, the following equivalence needed to be done because this pneumonia-without-VILI group never met the Berlin criteria for ARDS diagnosis. ARDS diagnosis timepoint in the pneumonia-without-VILI group corresponds to 30h since bacterial inoculum, which is the median time from inoculum to ARDS diagnosis in the pneumonia-with-VILI group.

Respiratory function

Arterial and mixed venous blood samples were collected every 6h to assess abnormalities in oxygenation with a PaO_2_/FiO_2_ ratio. Following a 3-second end-expiratory and end-inspiratory pause, respiratory system mechanics were calculated from ventilator parameters every 12h. We monitored peak, end-inspiratory and end-expiratory airway pressures, tidal volume, RR and minute ventilation. Respiratory system compliance, DP and mechanical power were derived from these data. Further, abnormalities in PaCO_2_ and minute ventilation were recorded as surrogate markers for dead-space ventilation. Lung vascular permeability was determined by extravascular lung water index (EVLW) via the PiCCO system.

Systemic and pulmonary hemodynamics

Every 12h, systolic, diastolic and mean pulmonary and systemic arterial pressures were recorded. In addition, we computed vasopressor dependency index defined as norepinephrine dose (µg/kg/min)/MAP (mmHg). Central venous pressure (CVP) and pulmonary artery occlusion pressure (PAOP) were also evaluated and used to calculate systemic and pulmonary vascular resistance. Cardiac output was obtained by thermodilution with Swan-Ganz catheter. Usual PICCO-derived parameters for preload, afterload and inotropism were also assessed. Additionally, every 12h, complete blood cell count, biochemistry and coagulation studies were carried out and reviewed, as displayed in **Table S2**.

Imaging, necropsy and histology

We examined lung infiltrate imaging via the thoracic X-ray at the beginning, ARDS diagnosis and end of the study.

Pigs were euthanized with 60 mEq of potassium chloride in one of the following conditions occurred: PaO_2_/FiO_2_ was less than 50 mmHg during two consecutive assessments, when pH <7.1 during two consecutive measurements or at the end of a 60-hour study post ARDS diagnosis.

At necropsy, the animals were positioned supine, and the lungs were excised, weighed and placed on sterile drapes. Tissue samples were taken from the most affected dependent and non-dependent region of each of the five lobes. This was done for histological, inflammatory, microbiological and permeability assessments, like lung/body weight ratio and lung wet-to-dry ratio.

Lung histology was evaluated with Hematoxylin-eosin staining. We classified the presence of mild, moderate or severe signs of diffuse alveolar damage according to extensions of 0-25%; 26-50% and >51% of the following parameters’ optic field: hyaline membranes, epithelial denudations, pneumocyte II proliferation; and intra-alveolar edema.

Systemic and local inflammatory markers

At baseline, pneumonia diagnosis, ARDS diagnosis and the end of study, we performed a bronchoscope-guided bronchoalveolar lavage (BAL) in the medium lobe to assess local inflammation. At the same timepoints, blood was drawn to measure systemic inflammatory markers. The obtained samples were processed as previously described (1). Interleukin (IL) IL-6, IL-8, were quantified by bead-based multiplex assays with Luminex technology (Millipore Iberica, S.A., Madrid, Spain).

Microbiology assessments

At baseline, pneumonia and ARDS diagnosis, and every 24h thereafter, we performed a BAL in the right lung for microbiological assessment. At the same timepoints, we collected tracheal secretions. Additionally, blood cultures were performed to detect *P. aeruginosa* bacteremia at the same timepoints and fever peaks. All microbiological samples including pulmonary tissue obtained upon necropsy were cultured. Bacteria were counted and identified, as previously described (1).

**REFERENCES**

1. Li Bassi G, Rigol M, Marti JD, Saucedo L, Ranzani OT, Roca I, Cabanas M, Muñoz L, Giunta V, Luque N, Rinaudo M, Esperatti M, Fernandez-Barat L, Ferrer M, Vila J, Ramirez J, Torres A. A novel porcine model of ventilator-associated pneumonia caused by oropharyngeal challenge with pseudomonas aeruginosa. Anesthesiology. 2014;120(5).
2. The European Committee on Antimicrobial Susceptibility Testing. Breakpoint tables for interpretation of MICs and zone diameters. Version 13.0, 2023 Available at: http://www.eucast.org (last accessed April, 2023).

**ADDITIONAL TABLES**

**Table S1.** Antibiotic susceptibility profile of *Pseudomonas aeruginosa* strain used in our experimental studies.

| Antimicrobial | Susceptibility by EUCAST (2) |
| --- | --- |
| Gentamicin | R |
| Tobramycin | R |
| Amikacin | R |
| Imipenem | R |
| Meropenem | S |
| Ceftazidime | R |
| Cefepime | R |
| Ciprofloxacin | S |
| Levofloxacin | S |
| Piperacillin/tazobactam | R |
| Aztreonam | I |
| Fosfomycin | R |
| Colistin | S |

MIC, minimum inhibitory concentration; CLSI, Clinical and Laboratory Standards Institute; EUCAST, European Committee on Antimicrobial Susceptibility Testing; S, susceptible; I, intermediate; R, resistant.

**Table S2.** Pneumonia diagnosis criteria.

|  | **Pneumonia with VILI**  **N=6** | **Pneumonia without VILI**  **N=4** | **p-value** |
| --- | --- | --- | --- |
| PaO_2_/FiO_2_  (Decrease from baseline) | -31.3±15.7 | -73.4±24.9 | 0.91 |
| Body temperature (ºC) | 38.6±0.5 | 38.6±0.4 | >0.999 |
| WBC (10^9^/mL) | 15.5±5.1 | 6.9±2.2 | 0.23 |
| Purulent secretions (%) | 100±0.0 | 75±0.0 | 0.44 |
| Tracheal secretions  (PA log CFU/mL) | 6.5±0.3 | 6.7±0.1 | 0.46 |

Data are reported as mean ± standard error. WBC, white blood cells; PA, *Pseudomonas aeruginosa.*

**Table S3.** Clinical data.

| Study time | **Baseline** | | **Pneumonia diagnosis** | | **ARDS diagnosis ^#^** | | **24 h** | | **48 h** | | **60 h** | | **P value** | |
| --- | --- | --- | --- | --- | --- | --- | --- | --- | --- | --- | --- | --- | --- | --- |
| Study group | **VILI** | **No VILI** | **VILI** | **No VILI** | **VILI** | **No VILI** | **VILI** | **No VILI** | **VILI** | **No VILI** | **VILI** | **No VILI** | **Group effect** | **Time effect** |
| WBC (10*9/L) | 16.1±1.1 | 12.1±1.5 | 16.6±6.0 | 6.9±2.2 | 20.9±2.8 | 18.5±3.9 | 26.0±3.5 | 21.5±3.5 | 21.7±7.3 | 15.53±0.28 | 16.3±5.5 | 15.19±0.52 | 0.092 | 0.080 |
| Platelets (10*9/L) | 389.5±30.2 | 429.0±34.4 | 251.8±26.1 | 303.3±60.6 | 194.8±22.3 | 214.3±23.2 | 167.2±12.7 | 206.3±14.9 | 227.0±15.1 | 311.3±48.2 | 228.3±18.1 | 364.3±60.7 | 0.077 | **<0.001** |
| Hb (g/dL) | 10.0±0.5 | 11.0±0.7 | 10.1±0.5 | 11.8±0.4 | 10.4±1.0 | 9.4±0.1 | 8.4±1.0 | 7.0±0.4 | 7.3±0.2 | 8.0±0.6 | 6.9±0.7 | 8.4±0.7 | 0.98 | **<0.0001** |
| Chlorine (mEq/L) | 103.5±1.1 | 105.5±2.0 | 108.7±2.1 | 104.3±2.2 | 108.2±2.8 | 106.8±1.4 | 109.5±3.3 | 106.5±1.9 | 101.5±4.1 | 83.8±24.6 | 98.0±4.2 | 107.8±1.8 | 0.64 | 0.24 |
| GGT (UI/L) | **70.0±8.3** | **25.0±6.4** | **36.0±2.8** | **26.0±5.8** | **37.5±2.3** | **14.5±3.4** | **43.0±5.3** | **17.3±4.3** | **38.8±4.5** | **13.6±3.5** | 38.0±5.8 | 15.1±4.9 | **0.002** | **0.002** |
| AP (UI/L) | 120.2±18.4 | 142.8±6.1 | 165.7±32.7 | 141.0±9.6 | 196.8±31.7 | 186.0±36.6 | 150.8±13.8 | 129.0±12.7 | 104.5±15.5 | 101.5±13.7 | 90.8±11.8 | 96.8±11.5 | 0.67 | **<0.001** |
| PT (sec) | 11.1±0.20 | 11.8±0.7 | 13.8±0.6 | 12.97±0.66 | 14.3±0.7 | 13.00±0.20 | 12.18±0.43 | 11.8±0.20 | 10.72±0.27 | 11.6±0.4 | 10.22±0.22 | 11.02±0.41 | 0.88 | **<0.001** |
| FG (mL/min) | 95.0±11.2 | 91.1±13.2 | 56.4±7.4 | 85.7±1.7 | 75.8±12.0 | 120.0±10.0 | **80.1±10.9** | **127.5±2.5** | 93.5±14.2 | 120.0±10.0 | 96.8±12.2 | 120.0±10.0 | **<0.001** | 0.11 |
| Creatinine (mg/dL) | 0.98±0.05 | 0.96±0.05 | 1.18±0.63 | 0.95±0.01 | 1.19±0.12 | 0.84±0.02 | 1.16±0.17 | 0.85±0.03 | 0.83±0.10 | 0.84±0.05 | 0.84±0.11 | 0.81±0.05 | **0.045** | 0.15 |
| Lactate (mmol/L) | 1.09 ±0.11 | 0.73±0.18 | 1.23±0.18 | 0.76±0.08 | **1.25±0.11** | **0.51±0.03** | **2.04±0.81** | **0.51±0.07** | 1.6 ±0.9 | 0.53±0.01 | 0.54 ±0.11 | 0.46±0.02 | **0.010** | 0.22 |

Data are reported as mean ± standard error. # ARDS diagnosis timepoint in only pneumonia group corresponds to 30h since inoculum, which is the median time from inoculum to ARDS diagnosis in the pneumonia-with-VILI group, and so forth. WBC, white blood cells; Hb, Hemoglobin; ALT, Alanine aminotransferase; GGT, Gamma-glutamyl transferase; PT, Prothrombin time; AP, Alkaline phosphates; GF, glomerular filtrate rate.

**ADDITIONAL FIGURES**

**Figure S1. Ventilator settings throughout the experiment.**

Pink solid lines and dots represent animals from the pneumonia-with-VILI group; and the green ones represent those animals from the pneumonia-without-VILI group. Data are reported as mean ± standard error of mean (SEM). # ARDS diagnosis time point in the pneumonia-without-VILI group corresponds to 30h from bacterial inoculum, which is the median time from inoculum to ARDS diagnosis in the pneumonia-with-VILI group. FiO_2_, fraction of inspired oxygen; PEEP, Positive end-expiratory pressure; RR, respiratory rate; TV, tidal volume.

**Figure S2. Fluid balance throughout the experiment.**

Pink solid lines and dots represent animals from the pneumonia-with-VILI group; and the green ones represent those animals from the pneumonia-without-VILI group. Data are reported as mean ± standard error of mean (SEM). **)** The mean evolution of vasopressor dependency index is displayed in pink and green dashed lines, respectively. # ARDS diagnosis time point in the pneumonia-without-VILI group corresponds to 30h from bacterial inoculum, which is the median time from inoculum to ARDS diagnosis in the pneumonia-with-VILI group.

**Figure S3. Macroscopic lung findings.**

**A)** The pneumonia-with-VILI group: All animals presented signs of severe pneumonia such as swelling, edema, hepatization. Some also presented pleuritis and pleural effusion. **B)** The pneumonia-without-VILI group: animals presented mild signs of pneumonia with less extended distribution.

**Figure S4. Histological score of pneumonia-with-VILI animals, gravity-dependence.**

Black boxes and whiskers represent dependent lobe areas while grey ones, non-dependent. Both are from the pneumonia-with-VILI animals. Data are reported as mean ± standard error. ATII, Alveolar epithelial type II cells.

**Figure S5**. **Inflammatory markers.**

Green boxes and whiskers represent the pneumonia-with-VILI animals while pink ones, animals without VILI. Data are reported as mean ± standard error. In the left section, interleukins measured in serum samples (A, C) are displayed and on the right, the ones measured in bronchoalveolar lavage samples (B, D). # ARDS diagnosis timepoint in the pneumonia-without-VILI group corresponds to 30h since bacterial inoculum, which is the median time from inoculum to ARDS diagnosis in the pneumonia-with-VILI group. This equivalence needed to be done for comparison reasons because the pneumonia-without-VILI group never met the Berlin criteria for ARDS diagnosis. * p<0.05

**Figure S1.** Ventilator settings throughout the study.

**Figure S2.** Fluid balance throughout the experiment.

**Figure S3.** Macroscopic lung findings.


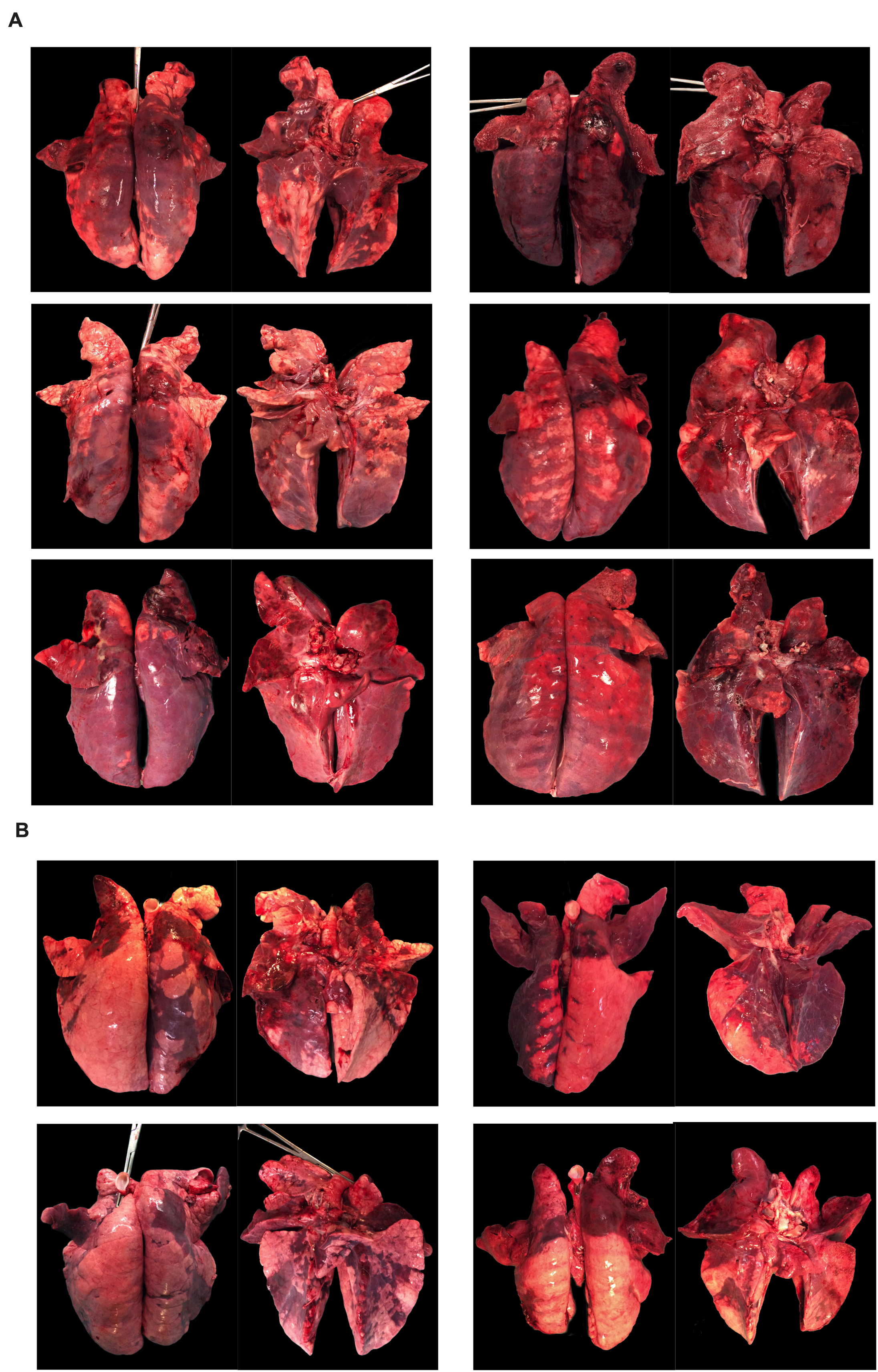


**Figure S4.** Histological score of the pneumonia-with-VILI animals, gravity-dependence.

**Figure S5**. Inflammatory markers.


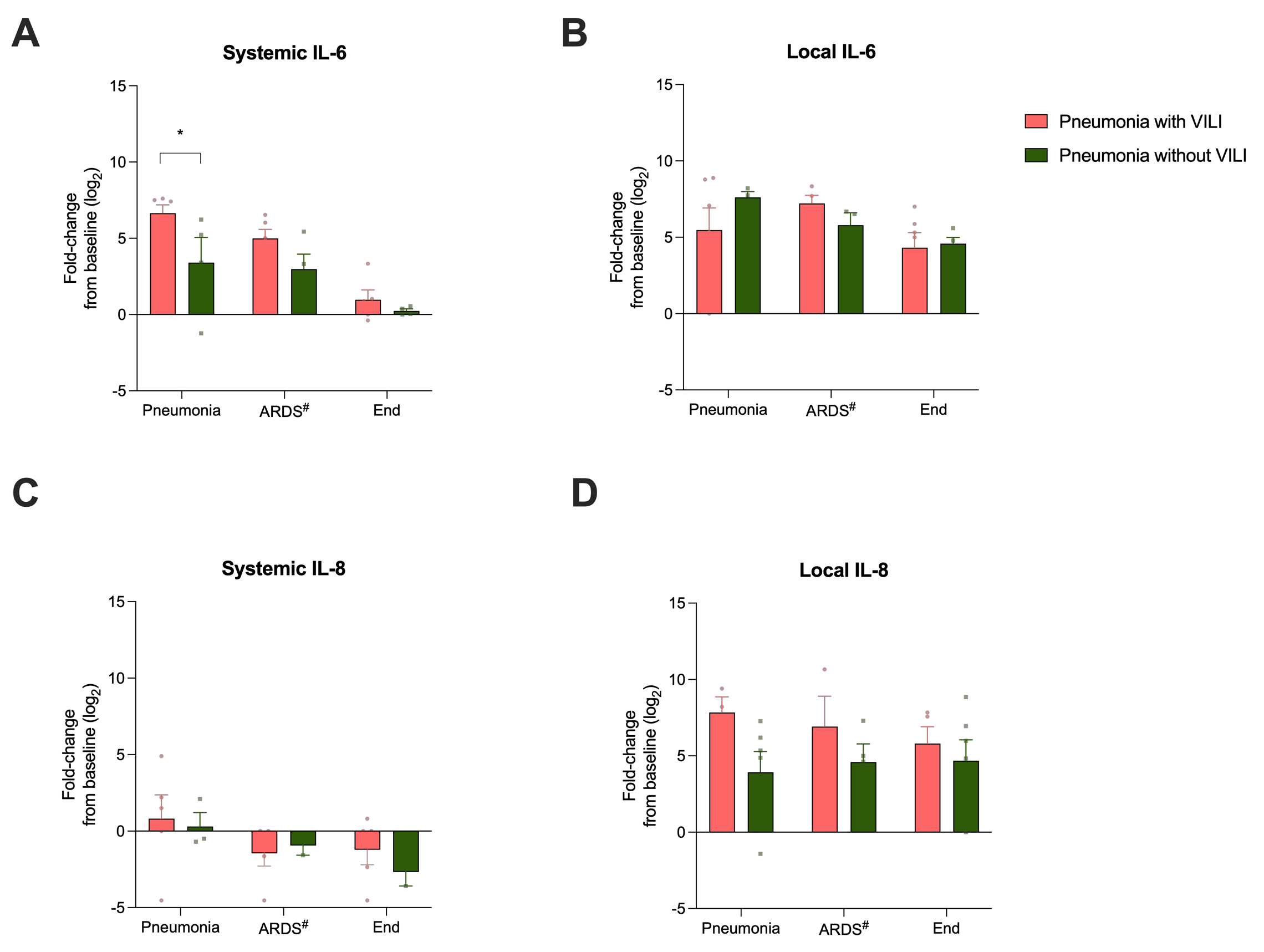

Supplement: Supplementary file 1 — Additional file 1. Additional methods, tables and figures. [file 13054_2023_4512_MOESM1_ESM.docx]
